# Structural Fatigue Life Monitoring with Piezoelectric-Based Sensors: Fundamentals, Current Advances, and Future Directions

**DOI:** 10.3390/s25020334

**Published:** 2025-01-08

**Authors:** Aliakbar Ghaderiaram, Erik Schlangen, Mohammad Fotouhi

**Affiliations:** Microlab, Faculty of Civil Engineering and Geosciences, Delft University of Technology, 2628 CD Delft, The Netherlands; a.ghaderiaram@tudelft.nl (A.G.); erik.schlangen@tudelft.nl (E.S.)

**Keywords:** fatigue life, monitoring, piezoelectric sensor, active sensors, passive sensors, dynamic loads

## Abstract

Structural fatigue can lead to catastrophic failures in various engineering applications and must be properly monitored and effectively managed. This paper provides a state-of-the-art review of recent developments in structural fatigue monitoring using piezoelectric-based sensors. Compared to alternative sensing technologies, piezoelectric sensors offer distinct advantages, including compact size, lightweight design, low cost, flexible formats, and high sensitivity to dynamic loads. The paper reviews the working principles and recent advancements in passive piezoelectric-based sensors, such as acoustic emission wave and strain measurements, and active piezoelectric-based sensors, including ultrasonic wave and dynamic characteristic measurements. These measurements, captured under in-service dynamic strain, can be correlated to the remaining structural fatigue life. Case studies are presented, highlighting applications of fatigue life monitoring in metals, polymeric composites, and reinforced concrete structures. The paper concludes by identifying challenges and opportunities for advancing piezoelectric-based sensors for fatigue life monitoring in engineering structures.

## 1. Introduction

Fatigue life monitoring (FLM) is crucial for safety critical structures, such as buildings, bridges, aircraft, and pavements, where failure due to cyclic or dynamic loads could lead to catastrophic consequences [1]. For example, around 85% of failures in metallic structures are caused due to fatigue [2]. FLM must be conducted using appropriate sensing technologies to assess the remaining lifespan of these structures, ensuring their safe operation and preventing downtime. These structures are made from various materials, including metals, fiber-reinforced composites, and reinforced concrete, each exhibiting unique fatigue-induced damage. As such, tailored approaches are required for the effective FLM and mitigation of fatigue failure. For example, in isotropic materials such as metals, the primary form of fatigue damage is the initiation and propagation of cracks [3] due to corrosion and loads. Microscopic cracks, often initiated at stress concentration points, can progressively grow with cyclic loading, leading to a decline in structural integrity. For fiber-reinforced composites, fatigue damage is mainly characterized by matrix cracking, delamination, and fiber breakage [4]. Matrix cracking occurs within the resin or matrix, compromising the composite’s load-bearing capacity. Delamination, the separation of layers, can result from repeated cyclic stresses, and fiber breakage can occur as the reinforcing fibers experience fatigue loading. In reinforced concrete structures, fatigue-induced damage encompasses microcracks, carbonation, chloride-induced corrosion, and delamination, leading to the initiation and propagation of larger cracks [5].

**Figure 1:**
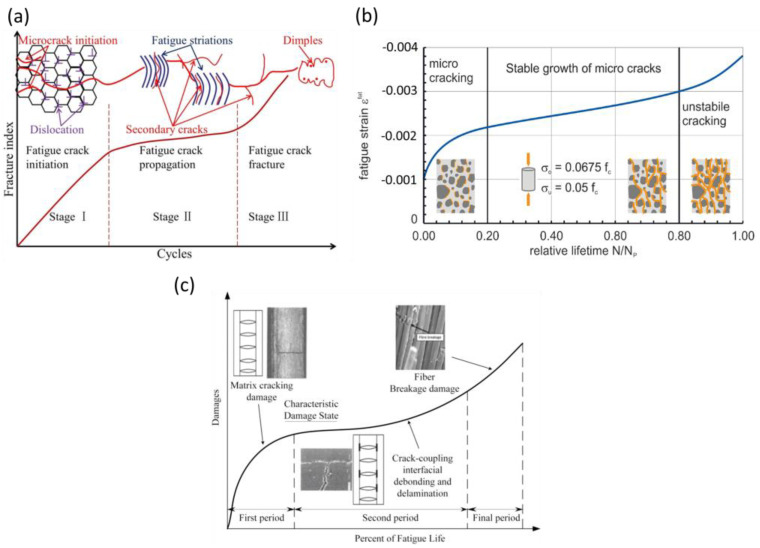
Fatigue-induced damage mechanisms in different engineering materials. (**a**) Damage evolution of a titanium alloy [6], (**b**) damage evolution in fiber-reinforced concrete [7], and (**c**) damage evolution of a polymer composite [8].

**Figure 2:**
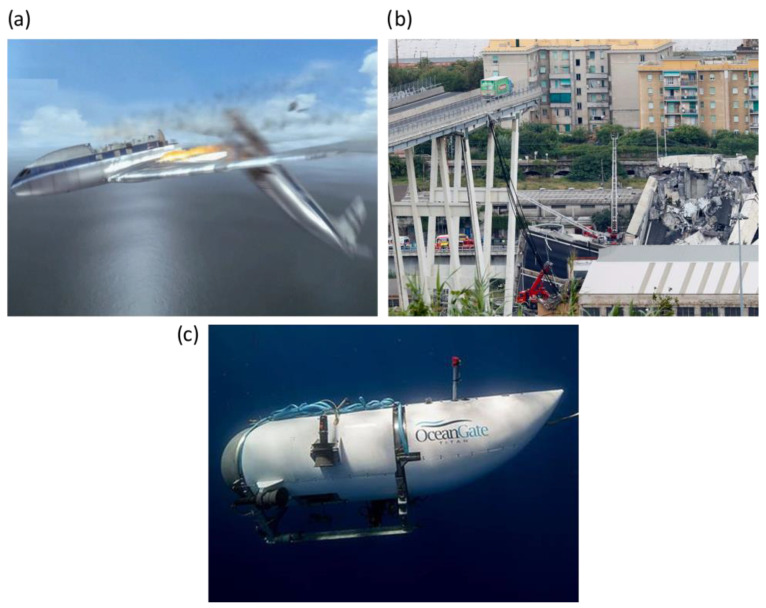
Examples of disastrous events caused by fatigue failure. (**a**) De Havilland comet plane crashes, metallic materials, 1954. (**b**) A highway bridge collapses, reinforced concrete, 2018. (**c**) Titan vessel crashes, fiber-reinforced composite, 2023.

As shown in Figure 1, different fatigue-induced damage mechanisms can compromise the structural durability of structures over time. Despite the different damage mechanisms, three different stages can be identified in fatigue failure: (i) damage initiation, (ii) stable damage propagation, and (iii) unstable damage propagation. FLM aims to detect the damage in stages I and II (see Figure 1), before it becomes critical. Failure to prevent fatigue failure can result in disasters, and examples are shown in Figure 2, where structures made of different materials failed suddenly, resulting in the loss of lives and high costs consequences.

There is a significant industrial demand for cost-effective and reliable FLM solutions to ensure safety, reliability, and longer service life. The current technology typically employs a range of sensors, including strain gauges, piezoelectric, electrical resistance, electromagnetic, and optical sensors, to measure parameters such as vibration, strain, load, and the size or shape of damage. These data can then be processed to detect changes and to identify patterns that are indicative of the remaining fatigue life of the structures, and can help in serviceability assessment and maintenance planning for structures [9].

Among the sensing techniques applied, piezoelectric-based sensors have been used widely for SHM purposes [10,11,12,13,14,15], due to their distinct advantages, including compact size, lightweight design, cost-effectiveness, flexible formats, a wide frequency range, high sensitivity to dynamic loads, and durability [16,17]. A fast dynamic response with high sensitivity, negligible decay, high fatigue life, and robustness under operational conditions characterizes them [18].

**Figure 3:**
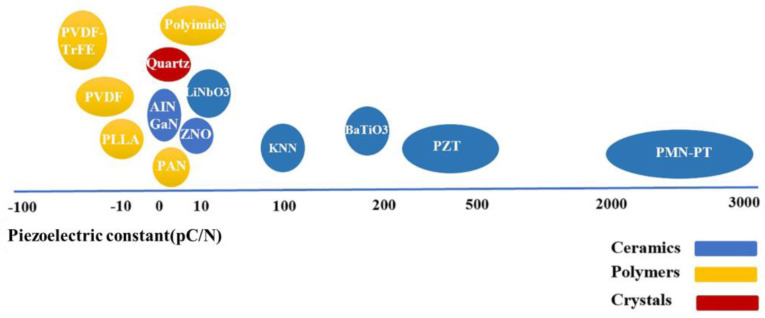
Comparison of the piezoelectric coefficient in different types of piezoelectric materials [32].

**Figure 4:**
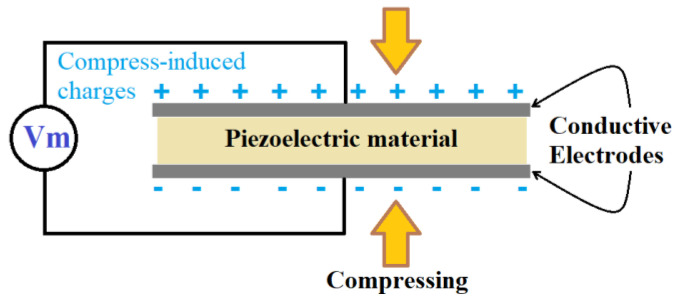
The piezoelectric sensor’s working principle under compression.

Piezoelectric-based sensors operate based on the piezoelectric effect, which is observed in certain materials, such as the ones shown in Figure 3. Lead zirconate titanate (PZT) and electroactive polymers like polyvinylidene fluoride (PVDF) are the most widely used materials. PZT is most employed in sensors and actuators due to its excellent piezoelectric properties (Figure 3). Although PVDF has lower piezoelectric coefficients than PZT, it stands out for its lightweight nature, flexibility, chemical resistance, and ease of processing [13]. Piezoelectric materials generate an electric charge when exposed to mechanical stress or pressure (see Figure 4) [19,20]. This property enables their use in various sensing techniques, including passive methods like the acoustic emission technique [10], and active methods such as the vibroacoustic modulation technique and the piezoelectric impedance method [21]. A detailed overview of the latest developments in piezoelectric materials and sensors for SHM is provided in a recent publication [22]. Several other review articles also discuss the application of piezoelectric sensors in SHM [16,19,20,23,24]. These reviews cover various applications, including polymer–matrix composites [21,25], aircraft [26], wind turbine blades [27,28,29], bridges [30], and other engineering structures [31].

This review paper provides an overview of the piezoelectric-based sensors for FLM.

## 2. Piezoelectric Sensor’s Application in FLM

**Figure 5:**
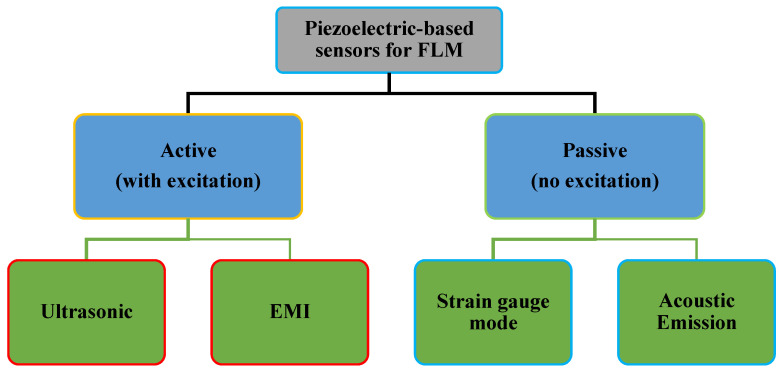
Comparative analysis of FLM techniques employing piezoelectric sensors.

**Figure 6:**
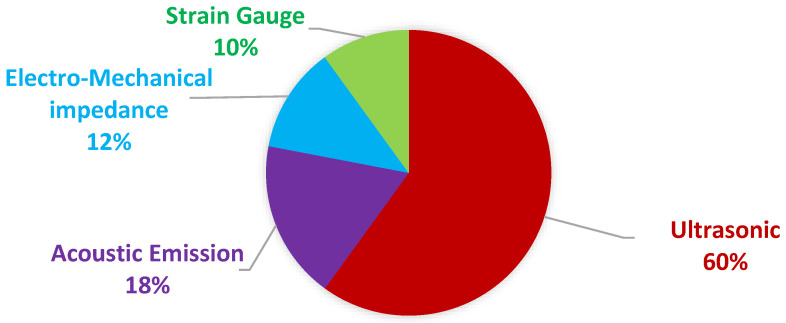
The proportion of piezoelectric sensor types used for FLM in the reviewed papers.

Figure 5 shows different piezoelectric-based sensors that are used in FLM. These sensors can be categorized as passive and active. Active methods include ultrasonic testing (UT) techniques, such as vibroacoustic modulation, which employs piezoelectric materials for Lamb wave generation and detection, and the electro-mechanical impedance (EMI) technique, which utilizes high-frequency structural excitation and impedance response monitoring. Passive methods include acoustic emission (AE) and strain gauge mode (STG) measurement techniques. These piezoelectric-based sensors monitor various parameters influencing fatigue life, such as crack or defect initiation, size, location, and dynamic strain history. Figure 6 illustrates the percentage utilization of piezoelectric-based sensors across active and passive methods for FLM among the papers reviewed in this research. UT is the most frequently used method, followed by AE, EMI, and strain measurement.

The following provides an overview of recent research on FLM using piezoelectric sensors, highlighting advantages, limitations, and categorized details of each study, including materials and methods, summarized in tables at the end of each section.

### 2.1. FLM Using UT

**Figure 7:**
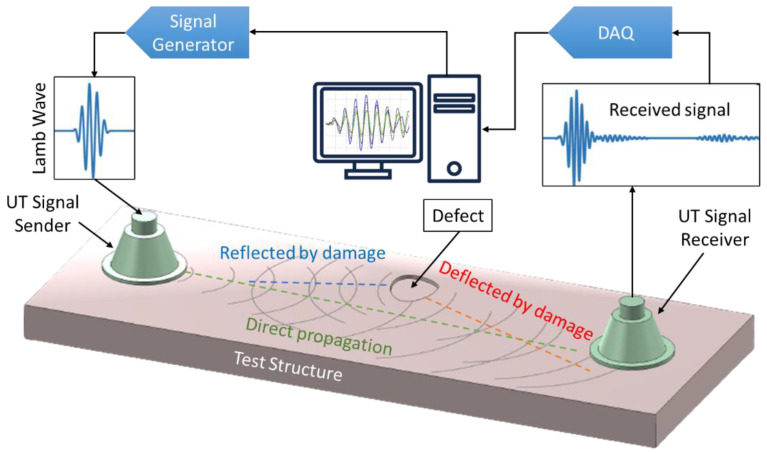
A simple demonstration of the UT NDT method principle in the pitch–catch mode.

UT-based crack detection is a commonly used technique for different types of materials [33,34]. It involves transmitting and receiving ultrasonic waves (from several tens of Hz to 1.5 MHz) to detect changes caused by cracks or defects [35]. The waves travel through the material and are reflected encountering a crack or defect. The reflected waves are then picked up by the same (pulse–echo method) or a different sensor (pitch–catch method), and their amplitude and time delay are measured [35], as depicted in Figure 7. The pitch–catch method utilizes low-frequency-guided Lamb waves (usually around 5 kHz to 100 kHz), which are effective for surface and subsurface defect detection due to their propagation characteristics and are less influenced by microstructural variations. However, this approach demands precise alignment and a complex positioning system. Additionally, analyzing the results can be challenging due to the multimodal and dispersive nature of low-frequency waves. In contrast, the pulse–echo method employs high-frequency waves, which are simpler to set up, requiring only a single sensor for transmission and reception. This method is cost-effective and easier to implement, but it demands a high voltage excitation and significant averaging to achieve a good signal-to-noise ratio and is more susceptible to interference from microstructural details and surface roughness. Both methods can detect transverse cracks and monitor their growth. The choice between these methods depends on the specific inspection needs, such as the type and location of defects, material thickness, and practical considerations like setup complexity and cost [36,37]. The ability of UT-based methods to detect cracks has been extensively reported in the literature. However, the sensitivity and accuracy of these methods depend significantly on various factors, including the type of structure, crack geometry, and the characteristics of the propagating wave. For instance, it has been demonstrated that cracks as small as 0.5 mm in length can be detected [38]. In another experimental setup utilizing the nonlinear features of Lamb waves, even smaller cracks with a length of just 1.35 μm were successfully identified [39].

Ihn and Chang [40,41] developed and validated a piezoelectric-based diagnostic technique for monitoring fatigue crack growth in metallic aircraft structures. Utilizing ultrasonic-guided Lamb waves, the method enhances sensor measurements and maximizes the signal-to-noise ratio for accurate damage detection. A physics-based damage index was used to correlate sensor data with crack growth size. Validation tests on notched aluminum plates and riveted fuselage joints demonstrated a strong correlation between the damage index and actual crack growth observed visually and using eddy current testing. These findings confirm the technique’s potential for accurate and reliable crack growth monitoring in complex structural applications, where cracks/debondings as small as 5 mm can be detected in riveted joints and composite repairs. In this research, Equations (1) and (2) were used to calculate damage index (DI) for critical crack length and deboned detection. S0 (symmetric zero-order) waves have symmetric particle motion and higher phase velocity, making them suitable for detecting subsurface and internal cracks. A0 (antisymmetric zero-order) waves exhibit antisymmetric motion, i.e., a lower phase velocity, and are sensitive to surface cracks and delamination [42].

**Figure 8:**
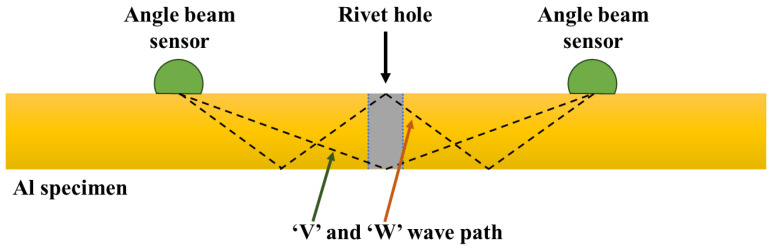
Schematic of the position of the UT sensors placed on the Al specimen.

In another study, Mi et al. [43,44,45] demonstrated the development and validation of an advanced ultrasonic method for the in situ monitoring of fatigue crack initiation and growth in aluminum specimens, specifically those with rivets and fastener holes. As shown in Figure 8, the method utilizes permanently mounted miniature angle beam sensors to detect changes in ultrasonic wave energy caused by the crack formation and modulated by the applied load. Two self-calibrating techniques were introduced: one compensating for sensor degradation using pulse–echo signals and the other using a normalized energy ratio of loaded to unloaded conditions, with the latter proving more effective for direct crack interaction measurement [43]. A “dynamic” measurement technique was also developed, allowing continuous monitoring during fatigue tests without interruption. This dynamic method accurately estimates uniaxial loads and correlates well with static measurements, demonstrating its effectiveness in tracking crack progression by modulating ultrasonic wave energy during load-induced crack opening and closing [44,45]. Theoretical analysis showed that the observed time shifts can be explained by changes in path due to elastic deformation and acoustoelastic effects. Further research is suggested to quantitatively assess these methods and determine tolerance levels for sensor and coupling degradation.

(1) $${DI}_{Crack\;detection}=\sqrt{\frac{Scattered\;energy\;of\;S0\;wave}{Baseline\;energy\;of\;S0\;wave}}\;$$

(2) $${DI}_{Debond\;detection}=\sqrt{\frac{Scattered\;energy\;of\;A0\;wave}{Baseline\;energy\;of\;A0\;wave}}\;\;$$

S. Gupta et al. [46,47,48], represented a real-time fatigue monitoring technique based on symbolic time series analysis (STSA) of UT data in an aluminum specimen. The optical images were captured every 200 cycles to investigate the uniformity distribution, and an analytical study of STSA for the obtained UT data, the anomaly profile, and the crack’s length were conducted. In order to analyze and process the UT data and investigate crack properties, P. Rizzo et al. [49] analyzed the fatigue crack process (crack initiation and propagation) of a steel beam, using a PZT sensor and actuator to emit and detect the ultrasonic-guided wave (UGW). An unsupervised machine learning algorithm and discrete wavelet transform (DWT) were utilized to probe the fatigue crack process and to calculate the DI. They concluded that the optimum testing frequency for the first anti-symmetric propagation mode was 225 kHz.

Experimental and finite element analyses were performed by C. Zhou et al. [50] for fatigue crack detection using nonlinear features of UT travelling waves. The authors used a network of permanently integrated active PZT sensors on an aluminum plate for quantitative crack monitoring. After the study on the influence of cracks on nonlinear features of UT waves, they developed a probability-based diagnostic imaging algorithm to visualize the results.

**Figure 9:**
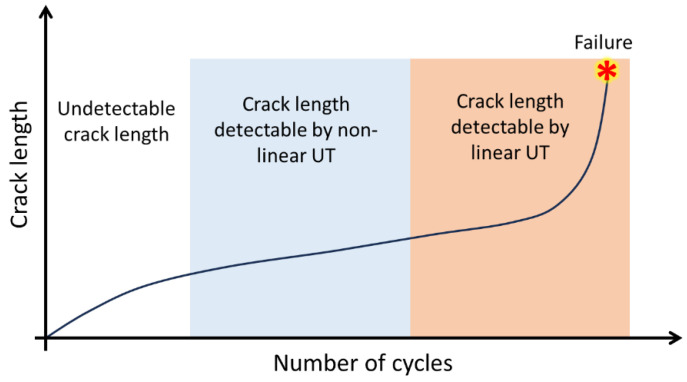
Comparison of linear and nonlinear UT measurements in FLM.

H. Cho and C. Lissenden [51] presented experimental and analytical research on fatigue crack monitoring in aluminum plates using UT. They used PZT discs as sensors to transmit and receive UT Lamb waves in the vicinity of fastener holes. They concluded that the transmission coefficient of propagated waves can be studied to detect and locate the fatigue crack. By comparing the transmitted wave amplitude before and after crack initiation, they achieved an FLM method in plate structures. Z. Su et al. [52] used UT wave features generated by four PZT sensors in the pulse–echo and pitch–catch modes for the quantitative evaluation of the fatigue DI in metallic plates. Comparing linear and nonlinear features of waves such as TOF and energy, they revealed that the nonlinear features of UT waves have a higher sensitivity than the linear features and it is more effective to detect small-scale fatigue cracks using nonlinear features. Figure 9 schematically shows the advantage of using UT’s nonlinear features, where the nonlinear measurements can detect much smaller fatigue-induced cracks/damage, and therefore provide timely information for effective maintenance and repair purposes. They also developed a probabilistic-based diagnostic imaging algorithm to show the crack’s location and size.

M. Hong et al. [53] conducted a theoretical model investigation for early damage detection using the nonlinearity features of UT. The modelling technique was developed to comprehend material, geometric, plasticity-driven, and contact acoustic nonlinearities that contributed to the nonlinear distortion of UT wave propagation. H. Chan et al. [54] evaluated the developed model in an experiment to monitor the fatigue crack around the fastener hole in a multi-layer aerospace 6061 aluminum plate using PZT sensors with high-frequency UGWs in the pulse–echo mode. A piezoelectric sensor was used to propagate and receive the Lamb wave. A non-contact laser measurement was also applied to evaluate the influence of fatigue crack in the aluminum layer on the Lamb wave scattering. They confirmed the feasibility of identifying concealed fatigue cracks in the second layer from a distance, without requiring access to the damaged specimen’s side, using a standard ultrasonic pulse–echo apparatus. P. Liu et al. [55] developed a wireless sensor node to monitor the fatigue crack using UT. They used two PZT discs to send two discrete high and low frequencies and received them by one PZT disc. Then, using the Discrete Fourier Transform (DFT) and considering the influence of occurred damage on the nonlinearity response of UT data, they investigated the statistical parameters “Skewness” and “Median” of the nonlinear index (NI). According to the NI, it was possible to detect the early-stage fatigue cracks without relying on any historical data of the target structure. In another study, M. Haile et al. [56] presented an analytical model using UT data to estimate fatigue crack growth in rotorcraft aerospace-grade aluminum structure and compared it with two industrial models, Paris–Erdogan and NASGRO. It was reported that the prediction error of the UT-based model was reduced by 50% compared to the two commonly used industry models.

A structural fatigue life prediction using Lamb waves was presented by D. Wang et al. [57], which focused on developing and validating data-driven models for crack quantification by experiments on coupon samples and lap joint tests. This study investigated the intricate relationship between model complexity, goodness of fit, probability of detection (POD) performance, and reliability in Lamb wave-based crack quantification for fatigue life prediction. The evaluation includes a nuanced consideration of the impact of the model choice on fatigue life prediction, emphasizing the need for a careful balance between model complexity and reliability in FLM. T. Jiang et al. [58] monitored fatigue damage in modular bridge expansion joints (MBEJs) in real time using PZTs. The approach involves analyzing the stress waves generated by the PZT sensor actuator and received by the PZT sensor. The experimental results showed that the amplitudes and wavelet packet energies of the signals received by the PZT sensor decreased as fatigue damage occurred. The proposed method can accurately detect the fatigue damage degree of any full-penetration weld in real time and has the potential to identify the initial fatigue damage occurrence in MBEJs. The authors verified the reliability and sensitivity of the proposed method using additional full-scale CB/SB assembly specimens with different fatigue loads. H. Jin et al. [59] introduced a reliable method to monitor fatigue cracks in SMA490BW steel plate-like structures using UGWs and various acoustic features. The proposed technique provides a hybrid DI by the fusion of DIs calculated using different acoustic features, such as amplitude-based and energy-based DIs in the time–frequency domain. The results demonstrated that the fused DIs calculated by the acoustic features in the frequency domain are more reliable than those in the time domain. Specifically, the linear and differential amplitude fusion DIs in the frequency domain are more promising to quantitatively indicate the propagation of fatigue cracks than other fused ones.

To improve the fatigue crack diagnosis (FCD) using guided waves (GWs), L. Xu et al. [60] used convolutional neural networks (CNNs) for signal processing to address the influence of dispersion on reliable FCD in real-world engineering applications. Using piezoelectric sensors to construct an input feature vector, the authors extracted multiple DIs from multiple GW exciting acquisition channels. A CNN was then designed to further extract high-level features from the multiple DIs and implement feature fusion for crack evaluation. The proposed method was validated through fatigue tests on a typical aircraft structure and showed promising results in reducing the influence of uncertainties on FCD. The lowest diagnostic accuracy achieved was 86.84%, with a diagnostic error of 1 mm. The authors also conducted comparative experiments that demonstrate the proposed method’s superior diagnostic robustness and accuracy compared to traditional methods. In another study [61], a new framework was proposed for quantitative evaluation and continuous monitoring of non-penetrating fatigue cracks in structures using a piezoelectric sensor. The framework consisted of a two-step process. In the first step, a 3D analytical model based on the theory of elastodynamics was used to generate contact acoustic nonlinearity in UGWs under the modulation of a non-penetrating fatigue crack, resulting in a crack-area-dependent nonlinear DI. In the second step, the 3D fatigue cracks growth model predicted the continuous growth of the identified fatigue crack in length and depth along the crack front. The framework was validated using numerical simulation and experiments, with the continuous prediction of the crack growth in length and depth. The results demonstrated the accuracy and precision of the developed modelling framework for characterizing propagating fatigue damage in real-life structures. The paper addressed the significance of characterizing and monitoring 3D, non-penetrating fatigue cracks at different stages of propagation, which are often simplified to two-dimensional models, risking evaluation accuracy.

Lamb waves that were actuated by piezoelectric sensors for fatigue crack detection and monitoring were also used to develop a two-dimensional cross-correlation imaging technique by W. Xiao et al. [62]. The imaging method is based on the cross-correlation algorithm that uses incident waves and crack-scattered waves of all directions to generate the crack image. The presented imaging method successfully inspects and quantifies the crack length and its growth. The use of scattered waves of all directions has the advantage of containing more information about the fatigue crack regarding the overall dimension of the crack. The frequency–wavenumber filtering method was utilized to extract the incident waves and the scattered waves. A scanning laser Doppler vibrometer was adopted for acquiring a time–space multidimensional wave field, followed by frequency–wavenumber processing. The proof-of-concept study was conducted on an aluminum plate with a hairline fatigue crack, and then, the imaging method was applied for crack growth monitoring on a stainless-steel plate undergoing fatigue loading. The paper suggested that the imaging method presented has the potential to be extended to different structural materials and more complex defects with highly irregular profiles. In another study, a new method for detecting and monitoring the growth of fatigue-induced cracks in welds was proposed by D. Zhou et al. [63] using piezoceramic sensors and coda wave interferometry (CWI). A theoretical model based on the acoustoelastic effect and CWI theory established a linear relationship between the crack width change and the relative velocity variation of coda waves. The authors conducted experiments using the CWI method to detect micro-fatigue cracks in three butt welded specimens under fatigue loading. The results showed that the relative velocity variation of coda waves increases linearly with the increase in the width of fatigue cracks. The CWI method was more sensitive to slight changes in welding fatigue cracks compared to the energy-based active sensing approach using a swept sine wave. The paper concluded by demonstrating the feasibility of the CWI method to monitor the slight variations in weld fatigue crack growth.

X. Zeng et al. [64] proposed an online updating strategy to accurately predict the propagation of fatigue cracks in an aircraft wing. The strategy combined an adaptively tunable hybrid radial basis function network and active Lamb wave-based SHM to capture the dynamic characteristics of piezoelectric sensor signals and predict the varying tendency of crack growth. The methodology employed a hybrid spatial-phase difference-based DI to quantify the crack length and deal with uncertainties during the prognosis of fatigue cracking propagation. The study validated the proposed method through fatigue tests on the outboard wing of a real airplane, demonstrating the effectiveness of the DIs method in mapping the varying tendencies of crack growth. The results showed that the posterior estimation of the crack length using the proposed methodology provides fewer measurement errors than the calculated values by the damage mechanics method. The proposed methodology has the potential to be expanded to other airframe structures and even the whole airframe. However, the early prognosis of crack growth results in errors and oscillations, which can be reduced by establishing a comprehensive model and a more effective DI method and real-life data collection as the focus of future research.

In another study, H. Lee et al. [65] developed a robust automatic damage diagnosis technique using UT Lamb waves and a deep auto-encoder (DAE) model that can accurately detect and classify fatigue damage in composite structures. The authors installed PZT sensors on carbon fiber-reinforced polymer (CFRP) composite plates to monitor fatigue damage evolution from matrix cracking to delamination. The collected UT signals were then used to train the DAE model, which effectively tracked UT response variations and diagnosed fatigue damage in the composite specimens. To enhance the accuracy and sensitivity of damage detection, the architecture and hyperparameters of the DAE model were optimized, and a statistical detection baseline was defined to capture damage indicators. The results demonstrated that the proposed technique can accurately detect and classify fatigue damage modes in a CFRP composite plate. It eliminated the need for manual or signal processing-based damage-sensitive feature extraction from UT signals for damage diagnosis. R. Nobile et al. [66] conducted an experimental investigation for monitoring the evolution of fatigue damage in carbon fabric open-hole specimens using UT measurements. The study showed that the UT signals acquired in wave packets with pitch–catch mode during the different phases of the fatigue life can be evaluated and correlated to the applied loads. The examination of the UT data revealed a high sensitivity of the UT signal to stiffness decrease and fatigue damage associated with delamination near the hole. Additionally, the consolidated pulse–echo phased array technique was used to evaluate the state of damage concerning the degradation of the signal detected with the PZT sensors. The results demonstrated the potential capability of the applied experimental technique for the real-time detection of delamination on composite elements subjected to time-varying loads. Furthermore, the study successfully applied an experimental procedure based on the UT propagation of Lamb waves to monitor in real time the onset and the progressive evolution of the damage up to failure.

**Figure 10:**
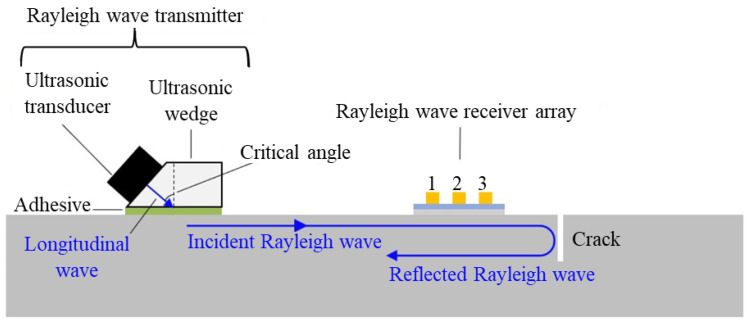
Schematic illustration of the system for monitoring the depth of a surface crack on a structure with Rayleigh waves [70].

To identify and quantify fatigue cracks in a steel joint under vibration conditions, Yu Lee and Ye Lu [67] utilized nonlinear GWs based on the second harmonic generation and piezoelectric sensors to excite the S_1_ Lamb wave mode in experiments. The contact acoustic nonlinearity was measured in experiments and quantified by a nonlinear index to evaluate fatigue crack. The study also involved a simulation using finite element analysis to verify the experimental results, and the outcomes were found to be in good agreement. The researchers proposed a percent reduction in nonlinearity as a measure to evaluate the crack length based on the difference of nonlinear index measured at static and vibration conditions. The results showed that the percentage reduction in nonlinearity increased with crack growth. The conclusion was that the proposed method is promising for fatigue crack identification and quantification based on second harmonic generation under practical working conditions of structures. However, further research is required to examine the exact contact area distribution between crack surfaces under the influence of applied vibration forces. In addition, for damage localization of complex structures, the nonlinearity caused due to ambient conditions, such as temperature and humidity variations, should be considered. An experimental investigation into the Lamb wave-based FLM of aluminum bolted joints with multiple sites was presented by C. Chen et al. [68]. The signals before and after the initiation of the fatigue cracks were compared to calculate the DI, which served as an indicator to map the fatigue crack size. The phase shifts between the baseline signal before the fatigue crack existence and the signal with fatigue cracks resulted in an overestimated DI. To solve this problem, the envelope damage index (EDI) was introduced to monitor fatigue cracks. The envelope curves of the baseline signal and the signal with fatigue cracks were considered in the calculation of EDI. The results showed that EDI is a better parameter than DI for monitoring the fatigue crack size and location in the aluminum bolted joints with multiple-site fatigue damage. The authors also validated the group velocity and time of flight for the S0 mode and the A0 mode and observed that fatigue cracks occur in hot spot areas. In the case of cracks in the vicinity of fastener holes in aircraft structures, V. Wong et al. [69] proposed a novel approach for detecting fatigue cracks, using direct-write piezoelectric UT sensors. These sensors were designed with an annular array of electrodes surrounding the fastener hole and made of PVDF piezoelectric material. The sensors could operate in both pulse–echo and pitch–catch techniques and could detect Lamb wave modes at a frequency of 1.5 MHz. The authors conducted numerical simulations and experimental testing to investigate the UT wave propagation and interaction with the defect. They used wavelet analysis and the energy ratio method to quantify the extent of the fatigue cracks. The results showed that the pulse–echo method could determine the direction of the fatigue crack, while the pitch–catch method had a higher sensitivity in crack detection but could not determine the crack direction. The direct-write UT sensors with annular array electrodes have a small footprint, lower cost, and compact size, making them suitable for the in situ SHM of fastener holes. Recently, X. Li et al. [70] presented a method for measuring surface cracks using Rayleigh waves and PVDF piezoelectric film UT sensor array (Figure 10). The method employed a delay-and-sum algorithm to enhance the detected Rayleigh wave signals and determine the depth of surface fatigue cracks. The proposed method was compared with Rayleigh wave detection using a Rayleigh wave receiver made of piezoelectric ceramic and a laser interferometer. The results showed that the low-profile PVDF film transducer array was more effective due to its low attenuation of surface-sensitive Rayleigh waves. The proposed method was suitable for monitoring crack initiation and early-stage propagation and is applicable to measure cracks present in complex structures such as welded joints. The crack depth monitoring procedure was successfully demonstrated by measuring fatigue cracks induced at two welded joints of a 38 mm thick steel cruciform structure under a cyclic mechanical loading test. The results indicated that the proposed method using a PVDF film UT sensor array is efficient and cost-effective compared to the laser interferometer and bulky piezoelectric ceramic Rayleigh wave receiver arrays. Table 1 presents a comprehensive overview of the literature, highlighting applications, monitoring strategies, and materials monitored using UT for FLM.

### 2.2. FLM Using AE

**Figure 11:**
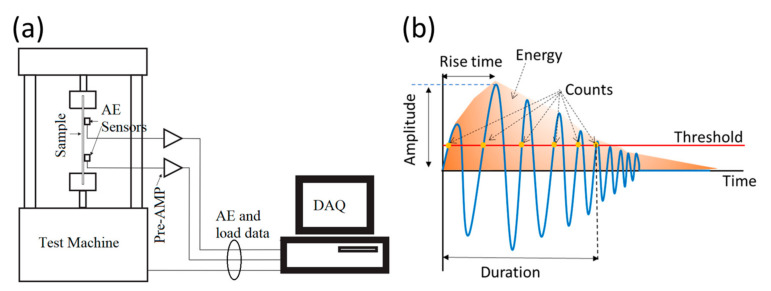
(**a**) A simple sketch of the AE experimental setup, and (**b**) a schematic of an AE waveform [80].

**Figure 12:**
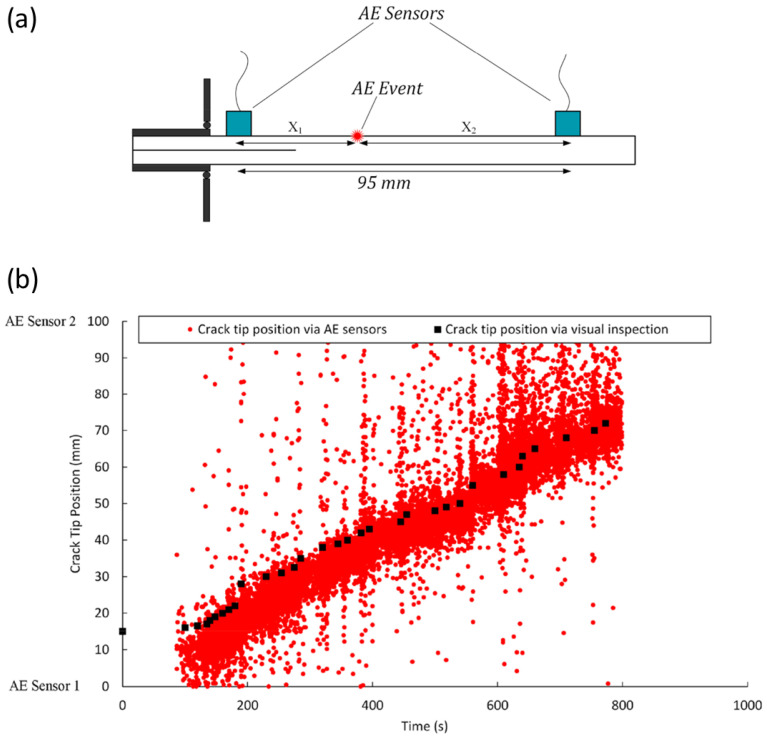
(**a**) The AE event localization procedures. (**b**) Prediction of fatigue-induced delamination size in laminated composites by AE [79].

AE is a passive non-destructive method that is used for the in situ SHM of engineering structures [71]. AE detects high-frequency acoustic signals generated by the release of energy from the material under stress (from several kHz to several hundred Hz) [72]. These signals can be detected using specialized AE sensors that convert the acoustic energy into electrical signals that can be analyzed and interpreted. The signals detected by AE sensors can be analyzed to determine the location and severity of damage in the structure. AE can also be used to monitor the progression of damage over time, as the signals generated by the release of energy from the material change as the size and shape of the damage evolves [73]. The AE technique is widely used for the detection and monitoring of fatigue cracks in metallic and composite structures. It is a sensitive and reliable method for detecting the onset of damage, as it can detect cracks that are too small to be visible using traditional inspection techniques, such as visual inspection or UT. But some limitations need to be addressed, such as environmental noises that originate from sources such as strong winds, passers-by, and trucks driving on nearby streets. These noise signals can be effectively mitigated by employing a bandpass filter [74]. Also AE signal propagation attenuation dependency on propagation distance limits this technique [75]. Since AE is affected by environmental noises, a false positive detection is likely which can be listed in the limitation list [76,77]. In AE testing, sensors are typically placed on the surface of the structure being monitored or embedded within the material itself (Figure 11). When the material undergoes stress or deformation, such as from a load or thermal expansion, the release of energy generates high-frequency acoustic waves that propagate through the material and can be detected by the piezoelectric AE sensors [78]. For example, Figure 12 shows the work by M. Saeedifar et al. [79] to determine the crack tip position during propagation of mode I delamination in glass/epoxy composite specimens. AE testing is considered a qualitative monitoring method, as it is difficult to quantify the damage level due to limitations such as the non-repeatable nature of the AE signals, sensitivity to noise, and the difficulty of interpreting complex signal patterns. Therefore, only very basic indicators, typically based on thresholds set on characteristic quantities of interest, are applied that can provide an estimate of the general condition, without detailed quantification of the extent of damage or damage topology. Although this offers some rough information, it should be improved to offer more quantitative information for monitoring purposes.

AE was used to monitor the health state of a flexible riser by T. Clarke et al. [81,82]. This riser contains several protective layers as well as helical wires in external armor. They measured strain over the wires using fiber Bragg grating was successfully used to validate the effectiveness of the AE sensor used on the riser circumference. When comparing the results from these two methods, they tried to relate the AE data to wire ruptures and load measurement. D. Gagar et al. [83] utilized AE to investigate the influence of fatigue loading and sample geometry on AE during crack growth in aluminum specimens. They studied the fatigue crack propagation and crack length by monitoring the change in AE Hit rates. Also, the location and length of the crack were investigated based on the AE event’s distribution. In another study, fatigue monitoring, using AE through PZT sensors, was conducted by M. Pearson et al. [84]. Three mapping techniques, time of arrival (TOA), Delta-t, and Akaike information criterion (AIC) Delta-t, were evaluated to estimate the fatigue damage location. Table 2 reports the average error of different techniques used for the source location, where the AIC Delta-t provided the most accurate estimation. They reported that using the AIC Delta-t mapping for locating damage in an SHM system would allow for the confident and more probable detection of damage irrespective of the threshold used.

AE monitoring and Bayesian estimation were used to detect and localize fatigue damage in girth-welded steel pipes by M. Shamsudin et al. [85]. The authors found a strong correlation between the AE energy and the estimated coefficients, which helped identify pipe cracks. The proposed method can potentially be used as an additional tool to increase confidence in source localization in AE testing and for monitoring crack growth in extreme conditions. The study highlighted the usefulness of AE monitoring for evaluating the condition of piping to manage the risk of vibration-induced fatigue failure. The study suggested further studies to validate the approach on a broader range of structural components and under various loading conditions.

Post-processing and the informative assessment of AE signals were conducted by J. Garrett et al. [86] to investigate the correlation between fatigue crack length and AE signal signatures. An automated AE waveform analysis technique was suggested using artificial intelligence methodologies, finite element analysis, and experimental investigation to capture and characterize AE waveforms. The experimental data were used with a CNN to build a system capable of predicting the length of the fatigue crack with an accuracy of 98.4%. The paper concluded that the proposed approach could be extrapolated to similar applications beyond binary crack length prediction and suggests that further experimentation with other crack lengths will be valuable in constructing a comprehensive crack monitoring system. In a recent study [87], the performance of piezoelectric patch acoustic sensors was compared to conventional AE sensors for detecting damage and leakage. The findings validate the potential of piezo for passive AE sensing, supporting their role as a complement to their well-established application in active sensing. Table 3 provides a detailed overview of the applications of AE for FLM in the literature.

### 2.3. FLM Using UT Waves and AE

A combination of AE and UT methods can also be used for FLM and detect associated damage and cracks. The two methods differ in their sensitivity and accuracy. AE is more sensitive to microcracks and is better suited for detecting early stages of damage [88], where the crack length is small and not detectable by UT, as schematically shown earlier in Figure 9, while UT testing is more accurate in determining the size and location of cracks [89]. Grondel et al. [90] conducted research to compare the result of the detection of cracks propagation in aluminum strap joint plates using a UT sensor and Lamb wave propagation, and AE method. X-ray images were also used to validate the AE and UT results, reflecting a good agreement between the AE and UT. M. Gresil et al. [91] simulated GW and AE for fatigue crack monitoring in a thick steel plate, and were able to calculate the DI and crack dimensions by taking advantage of the numerical method and wavelet-based signal processing. H. Mei et al. [92] utilized piezoelectric wafer active sensors (PWAS) as AE sensors for in situ AE detection during fatigue crack growth. The time of arrival of AE signals at multiple sensors confirmed the origin of the AE signals from the crack, and the amplitude decay of the AE signals was validated. J. Etxaniz et al. [93] developed an SHM Ultrasound System (SHMUS) that is capable of implementing both passive (AE) and active (UT) strategies to monitor structures for various types of damage, including sudden and progressive damages caused by impacts, fiber breakages, corrosion, delamination, and fatigue. The authors validated the SHMUS effectiveness on both metallic and composite structures and analyzed the effects of sudden and progressive damages on the integrity of the structures. The results showed that the SHMUS not only detected impacts and damages but also measured the structural integrity degradation and the damage increases in size. This developed SHMUS prototype was suggested for potential use in the aeronautical industry, as it can help monitor the integrity of aircraft structures throughout their entire life cycle. Table 4 provides a detailed overview of the applications of AE and UT hybrid systems for FLM in the literature.

### 2.4. EMI Measurements for FLM

**Figure 13:**
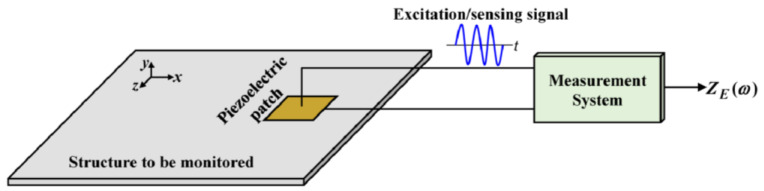
The underlying principle of the EMI method [97].

The EMI technique, originally introduced by Liang et al. [94], has been widely investigated for FLM. This method uses a piezoelectric sensor to excite the host structure while simultaneously recording the structural response. The sensor’s electrical admittance, which is the inverse of its impedance, is directly correlated with the mechanical impedance of the structure. As a result, changes in structural properties can be identified by monitoring variations in the sensor’s electrical impedance [95]. As shown in Figure 13, in EMI, a small alternating current, typically in the frequency range of kHz to MHz, is applied to the sensor. Subsequently, the resulting voltage, which is proportional to the sensor’s impedance, is measured [96]. EMI sensors can be strategically positioned at critical points on the structure to continuously track changes over time. The initiation and growth of small cracks cause measurable variations in the sensors’ impedance, enabling the assessment of the structure’s remaining fatigue life [19].

Soh and Lim [98] demonstrated the potential of using the EMI technique with surface-mounted PZT patches for detecting and characterizing fatigue damage in an aluminum beam. In a similar study, Li et al. [99] employed the EMI method for the real-time monitoring of fatigue crack initiation and propagation in an aluminum specimen. Their findings highlighted EMI’s high sensitivity to early-stage damage, successfully capturing the entire process of crack initiation, propagation, and unstable fracture under fatigue loading.

In another study, Giurgiutiu et al. [100] successfully demonstrated the pitch–catch approach to record the arrival time of the Lamb wave and EMI measurement at a higher frequency under fatigue loading, for crack growth detection in an Arcan steel. S. Bhalla et al. [101] used the equivalent stiffness identified by surface-bonded PZT patches, to quantify fatigue-induced damage in bolted steel joints and to predict the remaining useful life of the component. They conducted a comprehensive study on the effect of the host structure’s stiffness on PZT’s admittance. In this regard, they investigated the root mean square of the real part of PZT’s admittance (admittance (Y) = conductance(G) + j Susceptance (B)), as defined in Equation (3). Where $ReY_{i}$ is the real part of the electromechanical admittance of the PZT patch at any stage during the test, $ReY_{i}^{0}$ is the baseline value (in pristine condition), and ‘i’ represents the frequency index. The fatigue life was related to the equivalent stiffness and the remaining life correlated to the number of loading cycles.

(3) $$RMSD=\sqrt{\frac{\sum_{i=1}^{N}{\left(ReY_{i}-ReY_{i}^{0}\right)}^{2}}{\sum_{i=1}^{N}{\left(Re{ZY}_{i}^{0}\right)}^{2}}}$$

M. Haq et al. [102] used two embedded PZT sensors to detect mechanical movements and evaluate the fatigue life of a reinforced concrete column. The concrete column exerted cyclic vibration by a shake table, and then, a fast Fourier transform was applied to find and investigate the natural frequency of concrete column vibration. The results showed the natural frequency of the column was directly related to the structure’s stiffness and the natural frequency was decreased by increasing the damage. S. Bahalla and N. Kaur [5] used embedded piezoelectric sensors for monitoring low-strain fatigue-induced damage in reinforced concrete structures. The method involved piezo-based composite concrete vibration sensors (CVSs) embedded inside the beam near the surface, operating in the global and local modes. The experimental study demonstrated that the proposed method is effective in detecting and localizing fatigue-induced damage, and in predicting the remaining service life of the RC structure. The equivalent stiffness identified by the CVS in the EMI mode correlates well with the stiffness in the first and third regions of the fatigue S-N curve, making it suitable for detecting fatigue initiation and predicting final failure.

M. Haq et al. [103] utilized PZT-impedance sensors to monitor fatigue damage and assess the residual life of reinforced concrete frames, embedding PZT patches as actuators and sensors, and combining the EMI technique with global dynamic methods. Admittance signatures were acquired using six piezo-cement composite disks embedded at different locations in the structure. DWT, continuous wavelet transformation (CWT), and power spectral density analysis were applied to identify, localize, and estimate the severity of the damage. The proposed method was effective in diagnosing high-cycle and low-strain fatigue damage in reinforced concrete structures and is validated for all three phases of the fatigue life span of the structure. The rate of CVS-identified stiffness loss obtained after measuring equivalent stiffness parameters was found to be comparable in high-frequency fatigue loads, constituting 1.03 times higher stiffness change rate as obtained using actual flexural stiffness values of the RC structure.

In another study, life prediction models were proposed based on the residual stiffness, damping. and wavelet energy approach [104]. The DWT-based optimum methodology provided superior performance in enabling a real-time damage prognosis of reinforced concrete structures under low-strain and high-cycle fatigue. Two mathematical models were proposed for estimating the remaining useful life of reinforced concrete frames based on the equivalent flexural stiffness of the frame and the equivalent wavelet transform of the energy of conductance signatures. The study established the potential of the wavelet transform energy drawn using DWT signal processing techniques for characterizing damage and identifying the vicinity of beam-column joints as a critical area for fatigue failure. The method was found to be satisfactory in establishing a life-prediction model for the structure for any fatigue phase, which can be used to develop better collapse-alarming systems and prolong the structural life by retrofitting it to the appropriate location.

M. Haq et al. [105] proposed a novel application of wavelet to transform the energy of piezo-impedance signatures in monitoring the premature fatigue damage of reinforced concrete frames. The study included the implementation of the EMI technique combined with DWT on frequency domain PZT-admittance signatures to identify, localize, and quantify the severity of premature fatigue damages. The authors successfully developed residual-life predicting models based on EMI-identified equivalent structural damping and DWT-based wavelet energies in initial and severe stages to the deboning initiation. Additionally, the authors introduced an economical impedance-based solution using a miniature AD5933 chipset to monitor premature damages in externally retrofitted reinforced concrete structures. The proposed system can be further developed to create online wired or wireless monitoring systems utilizing different PZT forms for proposing electro-mechanical models for predicting the remaining life of different kinds of structures.

L. Ali et al. [106] used the EMI technique and finite element analysis for monitoring fatigue cracks in T-type joints of offshore steel jacket structures. The study highlighted the successful application of the EMI for the fatigue monitoring of the joints. Table 5 provides a detailed overview of the applications of EMI measurements using piezoelectric sensors for FLM in the literature.

### 2.5. Strain Gauge Mode for FLM

**Figure 14:**
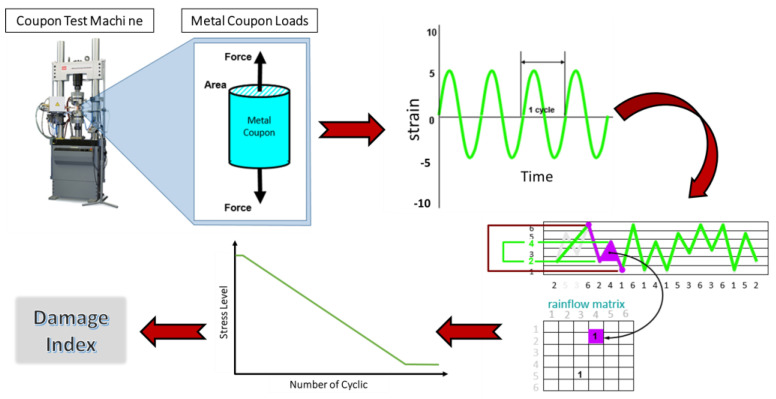
Strain profile measurement and its link with the S-N curves, considering the variation in the applied stress and frequency of the stress.

Piezoelectric sensors can be used as passive strain gauges for FLM by assessing the strains within a structure [107]. The strain measurement principle is based on the conversion of mechanical strain into an electrical charge by the piezoelectric sensor, thereby facilitating the measurement of dynamic strains over time which are responsible for fatigue failure [108]. Previous studies showed that there is a direct correlation between the strain level and voltage generated by piezoelectric sensors [107,109]. Figure 14 indicates the FLM principle using the strain monitoring concept, where the strain variation measurements (the severity and occurrence frequency) are used to measure the stress history of the material. The stress profile is then connected with the S-N curves, obtained from laboratory experiments, to evaluate the remaining life of the structure.

Aulakh and Bhalla [110,111] assessed the use of PZT patches for EMA-based structural identification and damage monitoring in a 2D rectangular steel plate. They found that strain modal parameters provided more sensitive damage features compared to accelerometer-based modal parameters, especially for detecting early-stage damage. In another study [18], piezo sensors were evaluated for modal identification of a pedestrian footbridge subjected to pedestrian motions, with results compared to accelerometer-based displacement modal parameters. The piezo sensors effectively captured low-amplitude dynamic strain responses, and strain modal testing showed a strong correlation with conventional acceleration testing. This approach was applied to real-life footbridges, where piezo sensors successfully detected bending and torsion modes, demonstrating high repeatability and minimal deviation.

Y. Zhang [112] used a paintable piezoelectric sensor to develop a method for measuring the surface crack on a metallic cantilever beam. The sensor was printed on the structure and was used to detect the crack(s) by comparing the electrodes’ output signal. The generated voltage was related to the DI by Equation (4), where $x1\left(t\right)$ and x2(t) are the measured voltage signals from each of the electrode pairs of the printed piezoelectric sensor, and RMS denotes the root mean square value of the measured voltage signal.

(4) $$Damage\;Index=\frac{\left[x1\left(t\right)-x2(t)\right]}{\left\{\frac{RMS(x1\left(t\right)+RMS(x2(t))}{2}\right\}}\;\;\;\;\;\;\;\;\;\;\;\;\;\;\;\;\;\;\;\;\;\;\;\;\;\;\;\;\;\;\;\;\;\;\;\;\;\;\;\;\;$$

N. Lajnef et al. [113] proposed a new approach by a combination of piezoelectricity and metal–oxide semiconductor (MOS) technology to make a self-powered and wireless sensor node for strain and temperature monitoring on pavements. They investigated MOS field effect transistors (MOSFETs) with floating gat to achieve a passive memory cell and to relate electrical parameters to temperature variation. They also studied the effect of traffic wander on fatigue life prediction.

A. Alavi et al. [114] represented distortion-induced fatigue crack detection methods in steel bridge girders, using a self-powered piezo-floating-gate sensor array. Piezoelectric sensors harvest the mechanical energy of crack displacement, and the generated energy is stored cumulatively to show the amount of crack displacement. Reading all floating gate cells in an array made it possible to localize the crack and its length. To determine the fatigue life, the J-integral concept and Paris Law were used. The results indicated that the proposed method is capable of detecting different damage progression states.

D. Kim et al. [115] utilized PVDF film sensors for fatigue damage monitoring of single-lap adhesive joints. The results showed that the PVDF film sensor exhibited constant sensitivity in terms of voltage per load, and the amplitude of the voltage signal increased as the maximum fatigue load increased. The applicability of the PVDF film sensor for fatigue damage monitoring was successfully demonstrated.

Other studies [116,117] used a piezoelectric strain sensor to measure the fatigue life of airframe structures, by measuring the crack closure, and the piezoelectric sensor outperformed traditional extensometers and back-face strain gauges in accuracy and consistency. The sensor reduced measurement scatter by at least 100%, with improvements nearing 200% under challenging conditions.

**Figure 15:**
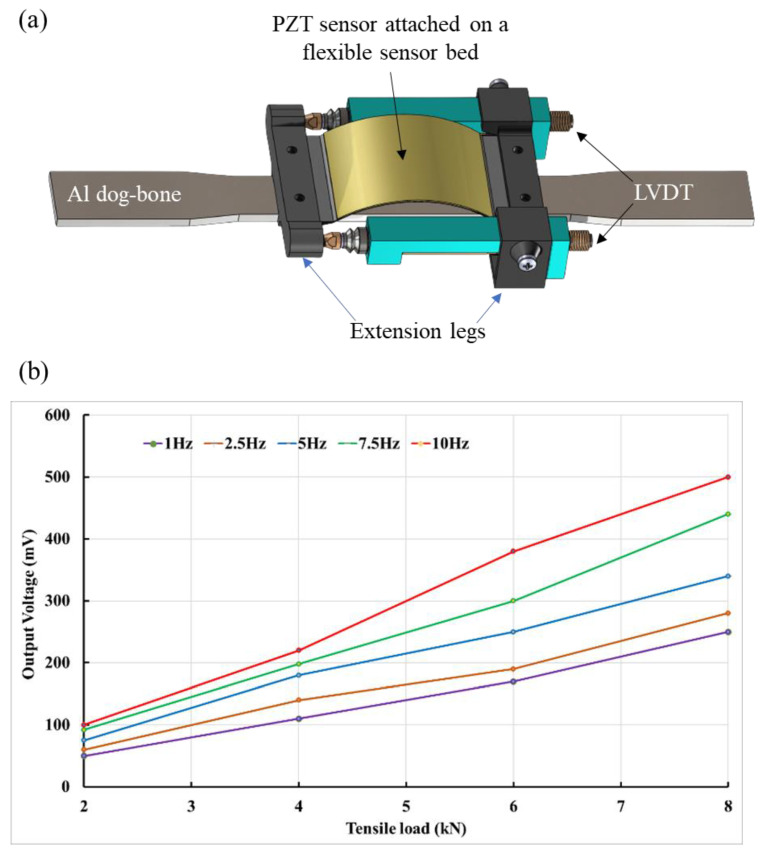
(**a**) Fatigue test setup schematic. (**b**) PZT sensor output in fatigue loading [108].

A. Ghaderiaram et al. [108] introduced an implementation strategy for using PZT in bending mode to measure strain, involving a 3D-printed extension bonded to the structure’s surface with epoxy glue. This extension offers several advantages, including the conversion of structural strain to PZT’s bending chord, preventing sensor rupture in high strain levels, and facilitating the measurement of one-dimensional strain due to the magnified voltage of PZT in bending compared to tension. Figure 15a illustrates the extension, featuring two legs that define the initial bending and a flexible sensor bed for sensor attachment. It also shows two built-in cases for LVDT installation, designed for sensor calibration. The preliminary results, presented in Figure 15b, demonstrate a linear relationship between the generated voltage and strain. As the tensile strain increases, the sensor output shows a corresponding linear increase in voltage, in line with the piezoelectric governing equation. Additionally, changes in loading frequency affect the curve slope. While these initial results are promising, further analytical development is needed to accurately quantify the PZT output as a function of strain.

Table 6 provides a detailed overview of the applications of strain measurements using piezoelectric sensors for FLM in the literature.

## 3. Signal Processing

The reviewed studies highlight diverse methodologies for processing sensor data. These approaches are tailored to specific applications, such as UT, AE, EMI, and strain measurement, and follow a structured framework that includes signal preprocessing, feature extraction and pattern recognition, data fusion (when multiple techniques are utilized), and subsequent quantification and statistical analysis.

Signal preprocessing is a foundational step reported in most studies. For instance, in UT-based applications, filtering techniques such as bandpass filters are employed to eliminate noise and isolate relevant waveforms [55,65]. Similarly, in EMI-based studies, baseline normalization is frequently used to correct for environmental fluctuations. Time-domain [40,41,43,44,45] and frequency-domain transformations, such as Fourier transforms [52,53,61,103,104], are also applied to enhance signal clarity.

The extraction of meaningful features from the processed signals is a critical step. For example, studies on strain measurement often analyze the amplitude and frequency of the piezoelectric output to detect deformation or fatigue-induced anomalies [108,113]. In EMI-based methods, changes in the resonant frequency or impedance spectrum are key indicators of structural damage [101,102]. In UT and AE applications, features like waveform energy, peak amplitude, and arrival time are commonly used to identify crack initiation and growth.

Advanced statistical and computational techniques are widely used to interpret the extracted features. Regression models and clustering algorithms, machine learning techniques, including neural networks [60], besides time-series and frequency-domain analysis methods, such as STSA [46,47,48], STFT [53], PCA [59], DAE [65], FFT [105], and Chio–Williams transform [86], have been used to monitor the evolution of features, providing insights into fatigue behavior. While the specific methodologies differ depending on the sensor type and application, a common trend is the increasing reliance on automated and computationally efficient techniques for data processing. Studies emphasize the importance of combining multiple features and processing steps to enhance accuracy and reliability. For instance, hybrid approaches integrating UT and AE methods have shown promise in improving the sensitivity of crack detection.

## 4. Integration Strategies

Integrating UT, AE, EMI, and strain measurement techniques can provide a comprehensive approach to fatigue life monitoring by leveraging the strengths of each method [118,119]. AE is highly effective for real-time active damage detection, especially for early warnings and identifying the onset and propagation of microcracks that may not yet be visible or detectable by other methods such as UT [120]. Complementing this, UT, an active sensing approach, excels in evaluating damage initiation, length, and dimensions, offering detailed insights into the size and morphology of damage [121,122]. EMI and strain measurement techniques fill a gap by providing information on the dynamic strain history of the structure, enabling the estimation of remaining fatigue life without requiring direct damage detection or damage size evaluation [117,123]. By combining these methods, a hybrid system can be developed where UT and AE are deployed for damage-centric inspections, while EMI and strain measurements are employed for global and local structural health assessment and fatigue life prediction. This integrated framework can enable enhanced reliability, early warning capabilities, and the potential for autonomous, data-driven fatigue life management across diverse structural applications.

## 5. Challenges and Opportunities for Future Research and Development

The literature highlights the promising potential of piezoelectric-based sensors for FLM, offering durability and high sensitivity to dynamic loading, essential for long-term monitoring under real-life conditions. However, challenges such as temperature sensitivity and susceptibility to electromagnetic interference need to be addressed during sensor design and data processing. Given the diverse mechanisms of fatigue-induced failure and material variations, monitoring solutions must be customized for specific applications, where parameters like damage level, crack length, local strain, or vibration play critical roles.

There is a significant gap in comprehensive research to develop cost-effective, reliable, and durable piezoelectric sensors for fatigue life measurement. More work is required to enhance sensor integration, understand long-term performance under varying environmental conditions (e.g., temperature, humidity), and develop low-power, flexible sensors. This will be key to fully leveraging piezoelectric technology for fatigue monitoring. Further research should focus on validating these methods under dynamic, time-varying conditions and explore multi-sensory systems that account for multiple parameters.

Future research and development in piezoelectric sensors for FLM should focus on the following:

- Sensor materials: Efforts should be directed towards developing high-performance, lead-free piezoelectric materials (e.g., BaTiO_3_, KNN) through advanced synthesis, characterization, and modelling to improve manufacturability, durability, and piezoelectric properties. Additionally, integrating flexibility and high piezoelectric performance into polymer-based composite materials is key to enhancing adaptability to complex structural loads. Improving sensor resilience to environmental factors such as temperature, humidity, and external loads, along with developing adaptive algorithms to mitigate these effects, is also crucial.
- Advanced manufacturing and embedded systems: Utilizing additive manufacturing and direct sensor printing to create customizable, scalable piezoelectric sensors. Focus should also be on improving the durability of embedded sensors for long-term use or developing easily replaceable alternatives to cover the structure’s lifetime. For example, civil structures may last over 100 years, and longevity is an important factor for FLM.
- Multi-sensor integration and systemic investigation: Transitioning from single-sensor to multi-sensor systems to improve accuracy and reliability. Combining piezoelectric sensors with other types of sensors and investigating fatigue damage, crack status, and environmental factors will help create integrated, cost-effective systems. The use of both active and passive monitoring techniques can provide a comprehensive damage assessment, while multifunctional sensors capable of both sensing and energy harvesting can enhance system efficiency and sustainability.
- Wireless and self-sustained systems: Incorporating wireless communication, self-powering mechanisms, and low-power operation to ensure ease of use and sustainability.
- Advanced algorithms: Research should focus on developing advanced algorithms for managing big data, utilizing machine learning and AI to improve signal processing, particularly in multi-sensor networks and real-life loading conditions for reliable monitoring.

## 6. Conclusions

Structural fatigue in engineering materials represents one of the most critical features determining structural integrity, which can lead to structural damage and collapse. Therefore, it is important to evaluate the fatigue-induced reduction in structural integrity, such as loss of stiffness and strength, or to measure fatigue-induced cracks/damage, and estimate the remaining fatigue life of the structure.

Among the available monitoring technologies, piezoelectric materials are widely used for FLM due to their durability, low cost, and high sensitivity for dynamic loadings. Overall, four piezoelectric-based sensing methods are used for this purpose, including UT wave propagation and reflection, in situ monitoring of AE signals related to active cracks and damage, measurement of the electromechanical impedance of sensors dependent on the structural integrity, and dynamic strain profile measurement by measuring the strain variations.

- UT can detect changes in the mechanical properties of the structure, as well as identify and locate existing cracks and damage at various stages of their development. This method is active and categorized into two modes, pitch–catch and pulse–echo, based on the arrangement of wave transmitters and receivers using piezoelectric sensors; each technique has its capabilities and weaknesses. The UT technique is well-suited for monitoring structures where prior knowledge of crack and damage status exists. It is particularly effective when cracks or damage can be monitored before reaching a critical length. However, its effectiveness is constrained when dealing with large areas that require continuous monitoring and limited information on the size and locations of cracks or damage. This limitation can be addressed through proper calibration and deploying an array of sensor nodes to cover various locations or through periodic UT inspections from different positions, although at an additional cost.
- AE is a passive method that captures the mechanical wave resulting from active crack/damage under the load, making it an ideal case for early damage detection. AE can also provide the location of active cracks/damage and provide rather qualitative measurements on whether those are in critical status or not. As a result, sometimes, a combination of AE and UT is used to take advantage of early damage detection of AE and accurate damage size measurements of the UT.
- In EMI methods, the measured electrical impedance of the piezoelectric sensor is related to structural integrity. In simpler terms, changes in the resonance frequency of the sensor reflect changes in the fatigue life of the structure. Impedance spectroscopy is considered an active method as it requires high-frequency electrical currents for measuring electrical impedance.
- Lastly, the strain sensing method using piezoelectric sensors, in addition to the accuracy and high-frequency response of piezoelectric sensors, provides a passive method capable of measuring dynamic loading-induced strains that are the main cause of structural fatigue failure and can be connected to fatigue life using S-N curves.

## Figures and Tables

**Table 1:** Detailed and categorized descriptions of the research in the field of FLM using UT waves.

Author(s)	Year	UT/EI/AE/ STG	Exp/Theor/Sim	Structure	Investigated Parameter(s)	Details
Ihn and Chang [40,41]	2004	UT	E/T	Aluminum plate-riveted fuselage joints	- Crack growth vs. no. of cycle. - TOF as a function of frequency. - Accumulated TOF for the multi-layer plate. - DI vs. the number of cycles for UT and EC techniques.	- Lamb waves were utilized. - An input signal in the range of 200–900 kHz, in the form of a windowed sine-burst wave, was utilized. - The highest signal-to-noise ratio (SNR) was obtained for the DI at about 420 kHz range. - Cracks as small as 5 mm in length can be detected.
Michaels et al. [43,44,45]	2006	UT	E/T	Aluminum	- Propagated signal amplitude. - Life expended. - TOF variation as a function of loading. - Transmitted energy variation during the number of fatigue cycles.	- Investigating single and double ‘V’ flying signal paths. - The double ‘V’ ΔTOF curve is least affected by the presence of cracks emerging from the fastener holes and, thus, is more accurate near the end of fatigue life.
S. Gupta et al. [46,47,48]	2007	UT	E/T	Aluminum	- A travelling optical microscope was used to measure the visible portion of the crack. - UT flaw detector. - Distribution of uniformity.	- Advantages of STSA for anomaly detection: (1) Robustness to noise and spurious signals. (2) Adaptability to low-resolution sensing. (3) Early anomaly detection capability. - Specimens used: 3 mm thick, 50 mm wide. - 12.5 Hz tension–tension loading applied. - For low-cycle fatigue: sinusoidal load, max/min loads at 92.5 MPa and 4.85 MPa. For high-cycle fatigue: max/min amplitudes at 71 MPa and 4.85 MPa.
P. Rizzo et al. [49]	2009	UT	E/T	Steel beam	- UT signal amplitude. - Crack extension as function a of cyclic load.	- The PSI-5A4E type PZT sensor was utilized. - Ranging frequencies swept between 50 and 300 kHz. - UT waves were processed using the DWT method. - An unsupervised machine learning technique was employed.
C. Zhou et al. [50]	2012	UT	E/S	Aluminum	- Phase and group velocity of Lamb waves. - Time–amplitude and frequency–time. - X-Y dimension visualization.	- An Al plate (380 × 400 × 4.5 mm) with four through-thickness rivet holes served as the fatigue target. - Study of the nonlinear relation between UT wave propagation in media and the effect of fatigue cracks. - 5 PZT were used for quantitative characterization. - A damage imaging algorithm was developed for visualizing and diagnosing damage.
H. Cho and C. Lissenden [51]	2012	UT	E/T	Aluminum plate	- Phase and group velocity UT waves. - Obstructed and diffracted wave amplitude variation during the crack growth. - Transmission coefficient variation as a function of crack length.	- A test structure composed of a 2-mm-thick 6061-T6 aluminum plate was used. - A sinusoidal cyclic load with a frequency of 5 Hz and a loading ratio ranging from 2.7 kN to 71.2 kN was applied. - PZT discs, 6.35 mm in diameter and 1 mm thick, were employed. - PZT transmitters received 350 kHz burst signals.
Z. Su et al. [52]	2014	UT	E/T	Aluminum plate	- Signal amplitude in the time domain before and after fatigue loading. - Frequency spectrum of signal under Gaussian white noise excitation. - Phase and group velocity of UT wave.	- A network of PZTs was utilized for generating and detecting UT waves in both pitch–catch and pulse–echo modes. - An aluminum plate with notches (484 × 300 × 2.2 mm) was subjected to a sinusoidal fatigue load of 4 kN 5 Hz. - Four PZT wafers, 6.9 mm in diameter and 0.5 mm in thickness, were employed. - A five-cycle Hanning-windowed sinusoidal tone burst at a central frequency of 300 kHz was used to investigate the DI.
M. Hong et al. [53]	2014	UT	E/S/T	Aluminum plate	- Group and phase velocity of Lamb waves. - Short-time Fourier transform (STFT). - Relative acoustic nonlinearity parameter.	- Two sources contribute to nonlinearity in the intact medium: material and geometric factors. - A modelling technique was developed using ABAQUS. - A 6061 aluminum plate (400 × 380 × 4.5 mm) was simulated.
H. Chan et al. [54]	2015	UT	E	Multi-layer aluminum	- Optical crack dimension measurement. - Laser interferometer scattered wave measurement. - Energy ratio of GW. - Reflected wave amplitude.	- 6.25 MHz pulse–echo GWs detected and monitored fatigue cracks. - Specimens consisted of two adhesively bonded 3 mm thick 2014-T6 aluminum plates (60 cm and 7 cm in length). - Maximum cyclic loading of 54 kN at 7 Hz was applied.
P. Liu et al. [55]	2016	UT	E	Aluminum plate	- NI of received signal evaluation using statistic “Skewness” and “Median” definitions.	- Three 0.5 mm thick, 18 mm diameter PZT discs were used. - The excitation unit produced two discrete frequency signals amplified to 28 V peak-to-peak through a 12-bit DAC. - Butterworth high-pass filter removed sub-2 kHz noise. - Wireless communication used a packed Z1 module. - Idle power consumption was approximately 105 µW. - Fatigue testing utilized a 6061-T6 aluminum plate under 10 Hz cyclic loading, with a maximum 25 kN load and 0.1 stress ratio.
M. Haile et al. [56]	2016	UT	E/T	Aluminum	- Crack length versus damage index. - Probability of detection vs. crack length, using UT. - Fatigue crack growth as a function of cyclic load, and comparison with Paris–Erdogan, NASGRO, and presented methods.	- The nonlinear dynamic state estimation problem presented in this study is described by a physical damage model (Paris–Erdogan law) and measurement model (UT sensor data). - An aerospace-grade Al7075-T6 aluminum alloy angle plates with a thickness of 1.6 mm were used as a fatigued specimen. - For the fatigue test, a constant load amplitude and aircraft rotor vibration were simulated in a loading range of about 15 kN. - UT pulses with center frequencies in the range of 0.1–15 MHz were transmitted through a material using piezoelectric sensors.
D. Wang et al. [57]	2018	UT	E/T	Aluminum coupon	- The normalized energy, phase change. And correlation coefficient. - Four data-driven models were proposed to correlate the crack size with damage-sensitive features.	- Aluminum coupon (2024-T3 alloy) measured 2 mm thick, 200 × 400 mm was used for the test. - Lamb wave applied at 160 kHz. - Two PZT sensors placed on either side of the crack in a pitch–catch configuration. - Fatigue crack propagation modelled using the classic Paris equation.
T. Jiang et al. [58]	2018	UT	E	Modular bridge expansion joints	- Influence of cyclic load on static load–displacement curve. - Effect of the number of load cycles on amplitude and energy of travelling wave.	- Dial indicators tracked beam vertical displacement during fatigue tests. - The PZT actuator received a swept sine wave from 0.1 to 150 kHz. - Cyclic loading involved a 2.5 Hz sinusoidal load ranging from 12 to 120 kN.
L. Xu et al. [60]	2019	UT	E/T	Aluminum	- GW signals. - CNNs. - Performance of the proposed GW-CNN approach for detecting and diagnosing fatigue cracks in aircraft structures. - Signal processing techniques such as time-frequency analysis and wavelet transforms for processing GW signals.	- The fatigue tests on attachment lug specimens in aircraft structures that are susceptible to fatigue cracks due to stress concentrations were the main subject. - Six attachment lug specimens made of 5 mm thick LY12 aluminum alloy with a hole diameter of 25 mm were tested. - The specimens were tested under a sinusoidal load with a peak value of Fmax = 18 kN, 10 Hz load frequency. - During the fatigue tests, a multi-channel PZTs array scanning system was employed to perform GW-based fatigue crack monitoring, using a three-cycle sine burst signal with a central frequency of 160 kHz and an exciting voltage of ±70 V.
H. Jin et al. [59]	2019	UT	E/T	Steel plate	- DI investigation based on the analysis of various acoustic features of the GWs, such as their amplitude and frequency content. - Compared the performance of several acoustic features and signal processing techniques in detecting and quantifying the damage caused by the cracks. - Potential applications of the proposed technique in real-world scenarios, such as SHM of aircraft and pipelines. - DI values calculation for the S0, S1, and A0 modes of the GW signals as a function of crack length - Principal component analysis (PCA) for the GW signals.	- The experiment was conducted on a plate-like SMA490BW steel specimen with dimensions of 2000 × 1000 × 10 mm. - A fatigue crack was introduced in the specimen by applying cyclic loading of 10 Hz with a stress ratio of R = 0.1. - The crack length was measured by a crack tip opening displacement (CTOD) gauge. - An active sensor network consisting of three pairs of piezoelectric sensors was used for GW excitation and reception. - The GW excitation frequency was set at 100 kHz. - Magnitude-based and energy-based DIs were constructed from the processed signals to quantitatively characterize crack growth along the actuator-sensor paths. - The effectiveness of the DIs was evaluated by comparing the DIs of different sampling points and different actuator-sensor paths in the time domain and frequency domain.
W. Xiao et al. [62]	2020	UT	E	Aluminum plate	- Crack imaging based on Lamb wave behavior in media. - A cross-correlation imaging technique was used to quantify and evaluate cracks using the incident waves and crack-induced scattered waves of all directions.	- The metallic specimens used in the fatigue tests were selected based on their mechanical properties and crack sensitivity and included aluminum 2024-T3 and stainless steel. - The specimens were cut using a vertical band saw to have a width of 100 mm and a length of 300 mm. - The highest load level was set at 65% of the yield strength, and the lowest level was set at 6.5%. - The frequency of applied cyclic fatigue loading was 4 Hz. - A hybrid system was configured using PZT for Lamb wave actuation.
D. Zhou et al. [63]	2021	UT	E/T	Steel plates	- CWI detects minor changes in a medium using multiple scattered waves caused by the existence of cracks. - The stretching coefficient is obtained by measuring the maximum cross-correlation coefficient between two coda waves. - The specimens were inspected using a visual inspection method and UT testing to confirm the presence of micro-cracks. - Wavelet packet energy analysis.	- The coda waves have better sensitivity to minor cracks due to their repeated sampling and amplification of small changes. - CWI theory obtains velocity variation in coda waves by measuring the time shift of coda waves at different times. - The welded specimens were made of low-carbon steel (Q235B) with a thickness of 8 mm. - The welding method used was gas tungsten arc welding with a welding wire diameter of 1.2 mm. - Fatigue loading was applied using a fatigue testing machine with a sinusoidal loading waveform with a range from 40% to 70% of the yield strength of the steel at a frequency of 10 Hz. - The CWI testing was conducted using a piezoelectric sensor with a center frequency of 2.25 MHz. - The experiments were conducted in a laboratory environment with a temperature range of 20–25 °C and a relative humidity of 40–60%.
L. Xu et al. [61]	2021	UT	E/S	Aluminum	- Nonlinear DI. - Offline phase predicts crack surface evolution via 3D fatigue crack growth model. - Crack-induced acoustic nonlinearity. - Crack-induced second source (CISS) introduced. - Higher-order wave modes of the regulated form of CISS determined via Fourier transform.	- Investigation of a two-step modelling framework, involving online and offline phases. - Crack surfaces’ “breathing” behavior was described as Crack Opening Displacement (COD). - PZT wafers (PSN-33, 8 mm diameter, 0.48 mm thickness) mounted on the aluminum 7075-T6 specimen surface for COD capture and calibration. - Pre-cracking cyclic load at 10 Hz, maximum 30 kN tensile load, and 0.1 stress ratio applied. - UT investigation was conducted using a Hanning-windowed 5-cycle sinusoidal tone burst at 250 kHz central frequency via the PZT sensor.
X. Zeng et al. [64]	2021	UT	E/T	Airplane’s wing	- Spatial phase difference. - Crack length. - Stress intensity factor range. - Statistical distributions of fatigue cracks. - Posterior probability density function of crack lengths. - Paris model. - High-definition cameras. - RMSE and MAE.	- Fatigue tests conducted on an actual airplane’s outboard wing. - Artificial notch created on rivet hole. - High-definition cameras are used to monitor crack propagation at the wing rib. - SMART Layer installed with 28 PZT-5A sensors (6 mm diameter, 0.33 mm thickness). - Actuating signals comprised five-peak sine waves.
H. Lee et al. [65]	2022	UT	E	CFRP composite plate	- Time domain UT responses compared for Lamb wave changes. - Amplitude and group velocity - UT signals entered optimized DAE model; RMSE evaluated reconstruction errors. - Damage-sensitive features from bottleneck used for fatigue damage mode classification.	- CFRP plates made with wet layup method, T700-SC-12K-50C fabric, and FSA23 resin in [0/90]s sequence. - PZT sensors (APC 841) enabled active sensing during fatigue tests, and a bandpass filter (30–90 kHz) was applied to signals. - Delamination damage sensitivity was observed more than matrix cracks in UT response changes.
R. Nobile et al. [66]	2022	UT	E	CFRP	- Amplitude and fundamental frequency. - UT signal displayed linear dependence on static load in tension–tension and tension–compression modes within specific load ranges.	- Specimens comprised different carbon/epoxy prepregs with twenty-layer laminates. - Set-up involved a signal generator, Control Unit, two 7 × 0.2 mm piezoelectric discs, and servo-hydraulic testing machine. - PZT transmitter received 300 kHz, 20Vpp input signal. - Fatigue tests applied tension–tension and tension–compression loading with 9 Hz frequency and stress ratios of 0.1 and −0.5. - Delamination caused initial signal amplitude reduction from 110.59% to 96%.
Yu Lee and Ye Lu [67]	2022	UT	E/S	Steel joint	- Acquired signals processed via FFT for time-to-frequency domain conversion. - Analysis focused on the first wave packet to reduce nonlinearity from other sources. - Second harmonic variations during vibration cycles confirmed damage. - Nonlinearity magnitude obtained by averaging three signals, each from 256 vibration cycles.	- Experiment on Grade 350 steel joint with twin 1 mm notches. - Applied stresses: max 150 MPa, min 15 MPa, at 10 Hz. - 10 mm circular PZTs formed a 4-path sensor network.
C. Chen et al. [68]	2022	UT	E	Aluminum bolted joint	- DI through before and after fatigue. - TOF. - Crack size calculation by DI and EDI.	- Bolted joint assembly fatigue specimen consisted of four parts. - Each specimen is mounted with twenty Smart Layer sensors.
V. Wong et al. [69]	2022	UT	E/S	Aluminum	- Dispersion curves. - Laser Doppler vibrometry. - Impedance analyzer. - Energy ratio. - Electromagnetic interference measurement.	- Ring-design piezoelectric UT sensor array proposed for focusing Lamb wave onto fastener hole center in pulse–echo or pitch–catch mode in 1.6 mm thick rectangular aluminum plate. - 1.5 MHz was chosen as the excitation frequency for dominant S0 mode Lamb wave excitation. - P(VDF-TrFE) powder used for fabricating the ring-design sensors. - STFT contour graphs indicated a narrowband UT signal, matching the excitation frequency.
X. Li et al. [70]	2023	UT	E	Steel cruciform	- Delay-and-sum algorithm. - Rayleigh wave detection.	- Rayleigh waves were generated using a UT sensor on a wedge with a critical angle. - PVDF film Rayleigh wave receiver array bonded in front of the crack, six sets on both surfaces of 38 mm thick S550 steel joint. - Three-point bending on top of the attachment plate, max 50 kN load at 3 Hz. - 50 μm thick PVDF film was used for the receiver array. - PVDF array compared with a laser interferometer and PZT-based array, unable to measure defect depth due to wave attenuation and low damping. - Rayleigh wave array tracked crack depth during fatigue cycles, constrained by −6 dB frequency bandwidth of incident wave.

**Table 2:** Summary of the average Euclidean error in the source location [84].

		Average Euclidean Source Location Error (mm)		
		TOA	Delta-t	AIC Delta-t
Aluminum specimen	N-H sources	32.6	5.8	3.0
	Fatigue damage	20.2	4.2	3.4
Composite specimen	N-H sources	19.3	18.9	4.2
	Impact events	124.7	18.9	3.3
Aircraft panel	N-H sources	13	8.7	3.6

**Table 3:** Detailed and categorized descriptions of the research in the field of FLM AE.

Author(s)	Year	UT/EI/AE/ STG	Exp/Theor/ Sim	Structure	Investigated Parameter(s)	Details
D. Gagar et al. [83]	2015	AE	E/S	Aluminum sheet	- Fatigue crack growth rates versus crack tip stress intensity. - Average AE Hit rate and coefficient variation. - Probability of AE Hite occurrence as a function of crack length.	- Fatigue test on 2014-T6 aluminum. - Specimens underwent constant amplitude loading at 2 Hz and stress ratios between 0.1 and 0.5 (stress ranges between 27 and 52.2 MPa). - Kernel Density Estimation represented AE hit intensity for different crack lengths and fatigue cycle positions. - AE rates peaked for 10–20 mm cracks around the mean stress cycle of approximately 32 MPa (15–35 MPa).
T. Clarke et al. [81,82]	2011	AE	E	Flexible riser	- FBG strain measurement. - AE events. - Displacement and torsion angle during time and investigating wire ruptures.	- AE and FBG methods were used to monitor helical wires’ rupture in riser armor. - 4 AE sensors fixed at 90-degree intervals around the riser. - A 0.01 Hz cyclic load ranging from 150 to 2100 kN was applied.
M. Pearson et al. [84]	2016	AE	E	Aluminum and composite structure	- Arrival time of AE. - Estimation of pencil-breaking test location. - Events located throughout the fatigue testing.	- 2024-T3 Aluminum (370 × 200 × 3.18 mm) was fatigued. - AE signal recorded with a 40 dB threshold, 100–1200 kHz filters. - 2 Hz fatigue loading, 0.25–24 kN range was employed for the test. - The AIC method demonstrated superior precision for impact event localization, with an average error of 3.3 mm.
M. Shamsudin et al. [85]	2019	AE	E	Welded steel pipe	- AE signals. - Signal length and its effect on Bayesian estimation. - Crack size estimation using Bayesian estimation. - Differences between signals generated by PLB and actual crack signals.	- Resonance fatigue testing was performed on a 381 mm diameter, 50 mm wall thickness, and 7.2 m long carbon steel pipe. - PLB tests conducted with signal lengths from 50 μs to 500 μs, measuring elastic wave time of flight between two sensors. - Bayesian estimation utilized located AE events and prior distributions from PLB tests.
J. Garrett et al. [86]	2022	AE	E/S	Aluminum	- Crack growth is continuously tracked using a camera and eddy current equipment. - Choi–Williams transform applied to the wave for processing of AE signals. - AE hits.	- A 1 mm thick aluminum 2024-T3 with a 4 mm crack was tested. - PWAS, S9225 sensors, and non-reflective boundary monitored AE. - Loading ranged from 1.38 kN to 13.85 kN at 10 Hz to induce a crack. - AlexNet CNN predicted crack length from AE signals.

**Table 4:** Detailed and categorized descriptions of the research in the field of FLM using UT waves and AE.

Author(s)	Year	UT/EI/AE/STG	Exp/Theor/Sim	Structure	Investigated Parameter(s)	Details
Grondel et al. [90]	2002	UT/AE	E/ S	Aluminum strap joint	- X-ray investigation. - Phase and group velocity dispersion. - and excited as a function of frequency. - Arrival time of the Lamb wave. - AE event.	- The important keys in this test include sensitivity and propagation distance. - The dispersion of the S0 and A0 modes was 800 Hz.mm, and the excitation above 2 MHz.mm would result in the generation of other modes. - A low-thickness ceramic Piezoelectric, P1-60, a standard “Quartz and Silice”, was used for manipulating Lamb waves. - In the AE method, a PACR-15 resonance sensor (150 kHz) and a narrowband filter were employed.
M. Gresil et al. [91]	2011	UT/AE	E/ S	Thick steel	- Phase shift and amplitude change in UT waves. - Transmitted and scattered waves through the media. - DI simulation. - Acoustic events. - Pencil break AE. - Crack localization.	- Signal processing and Lamb mode identification were performed using the Daubechies wavelet transform and FFT analysis. - A comparison between conventional AE sensors and PWAs was conducted for an AE pencil break test. - Active sensing in pitch–catch mode was used for DI calculation. - Fatigue tests were performed under a load-controlled mode with a frequency of 1 Hz and tension load ranging from 0.5 to 50 kN.
H. Mei et al. [92]	2019	UT/AE	E	Aluminum plate	- EDS and SEM analysis were conducted to obtain chemical compositions and visualize the microstructure. - Time–space Lamb wave data were obtained from the SLDV line scan, and the experimental frequency–wavenumber dispersion curves were obtained. - An SLDV area scan was conducted to measure the wave interaction with the corrosion damage under PWAS excitation. - Experimental tuning curves showed that the S0 mode was dominant around the 330 kHz frequency in the 0° direction, while SH0 mode was also observed as strong as S0 mode in the 45° direction due to the anisotropic behavior of the composite plate. - The pitch–catch mode showed that both S0 and SH0 were dominant in the sensing path, in agreement with the experimental tuning curves.	- The circular PZT with a diameter of 7 mm and a thickness of 0.2 mm was used. - PWAS was studied for detecting damage in isotropic plates, particularly on aluminum plates using circular and long PWAS. - A simulated corrosion damage was made on the aluminum plate to study damage detection using the active sensing method. - Lamb waves were generated in the aluminum plate under electrical excitation, and mode conversion and scattered waves were picked up by a Polytec PSV-400-M2 SLDV. - A network of PWAS sensors and damage imaging methods were used to detect and quantify delamination. - Signals were collected using pulse–echo and pitch–catch modes. - The direction-dependent group velocities of the incident and scattered waves for all the individual sensing paths on the composite plate were determined using the semi-analytical finite element analysis. - Based on the TOF of scattered SH0 waves, the imaging method was able to detect and quantify the delamination in the plate.
J. Etxaniz et al. [93]	2023	UT/AE	E	Composite and metallic	- Comparative study between isotropic (metallic) and anisotropic (CFRP) materials. - Passive testing for impact energy and position detection. - Ultrasound wave.	- SHMUS prototype capable of simultaneous signal generation and acquisition for 18 channels. - PWAS set utilized for UGWT (5–20 units). - Integrated Impact Detection System within SHMUS. - Waveform processing is executed by an internal FPGA logic circuit.

**Table 5:** Detailed and categorized descriptions of the research in the field of FLM using the piezoelectric sensor for EMI.

Author(s)	Year	UT/EMI/AE/ STG	Exp/Theor/Sim	Structure	Investigated Parameter(s)	Details
Giurgiutiu et al. [100]	2006	EMI	E	Galvanized mild Arcan steel	- Real part of EMI as a function of frequency. - Arrival time of the Lamb wave.	- The frequency range of the EMI signature was 100–500 kHz. - The pitch–catch approach uses a UT actuator with a 3-count sine tone-widowed sine burst in the range of 474 kHz. - The maximum crack size was 17.3 mm at 94,000 cycles.
S. Bhalla et al. [101]	2012	EMI	E/T	Bolted steel joints	- PZT’s conductance (G)-frequency. - PZT’s susceptance (B)-frequency. - Root mean square deviation (RMSD) of PZT’s admittance. - Stiffness looseness during cycles of loading.	- A comprehensive and formulated explanation of PZT impedance was presented. - Research was conducted using double-lap shear joints. - Loading cycles ranging from 3 to 5 Hz were applied.
M. Haq et al. [102]	2017	EMI	E	Reinforced concrete column	- Shift in natural frequency of concrete beam during the cyclic vibration.	- A 1.4 m long and 0.15 × 0.15 m cross-section reinforced concrete was used to conduct experiments.
S. Bahalla and N. Kaur [5]	2018	STG/ EMI	E/T	Reinforced concrete	- The RMSD and conductance of PZT’s admittance. - Beam stiffness vs. EMI signature. - DI was studied.	- 10 × 10 mm PZT sensors embedded in concrete served as both dynamic strain and EMI sensors. - Cyclic vibration applied via a 75 N, 17.2 Hz shaker. - EMI measured using an LCR meter in two ranges: 5–250 kHz and 0.1–1 MHz.
M. Haq et al. [103,104]	2020	EMI	E/T	Reinforced concrete	- The RC frame’s free vibration response was measured using CVS-F as a dynamic strain sensor. - Natural frequency. - RMSD index. - Fatigue damage location. - Exploration of damage states. - Mathematical relations for equivalent values and normalized DI. - EMI technique-based conductance signals processed using DWT and CWT in the frequency domain.	- Six PZT patches in CVS form captured electro-mechanical response in the d31 mode for flexural vibrations. - 1:4 scale RC frame simulated real-life conditions using M20-grade concrete and Fe415/Fe250-grade steel. - GDT and EMI techniques concurrently monitored RC specimens under high-cycle fatigue loads using CVS for global and local damage assessment. - Global dynamic technique found natural frequency through impact test and shake table excitation. - De-noising effectiveness shown in a comparison of original and de-noised conductance signatures for CVS (CR = 0 and CR = 1). - De-noised G-signatures depicted for different damaged states in the 100 kHz to 200 kHz excitation frequency range using CVS-F. - Daubechies wavelet (DB2) of order 2 is used as the mother wavelet function for both DWT and CWT.
M. Haq et al. [105]	2022	EMI	E/T	Concrete frame	- FFT of PZT signal. - Conductance signatures. - Wavelet energies of conductance signatures.	- One-bay, one-story damaged RC portal frame retrofitted with steel plates and CFRP wrap using Sikadur adhesive. - RC specimen was excited with sinusoidal cycles. - Five PZT patches were utilized, with four surfaces bonded to steel plates, one on CFRP wrap, and one embedded in concrete. - GDT and EMI responses were recorded using a DSO and LCR meter with a miniature impedance chipset. - GDT was used to determine the structure’s natural frequency. - “The Kelvin–Voigt system” was used to demonstrate meticulous structural damages.
L. Ali et al. [106]	2021	EMI	E/S	Offshore steel structure	- Fatigue Damage Location.	- Offshore structure fatigue strategies based on S-N curves from tubular joint testing. - T-type plate Joint fatigue tested at 1 Hz frequency and 150 kN magnitude to induce cracks within a limited timeframe.

**Table 6:** Detailed and categorized descriptions of the research in the field of FLM using the piezoelectric sensor as a strain gauge.

Author(s)	Year	UT/EMI/AE/STG	Exp/Theor/Sim	Structure	Investigated Parameter(s)	Details
Y. Zhang [112]	2006	STG	E	Steel cantilever	- Strain test under vibration on piezoelectric paint sensor and metal foil strain gauge. - DI before/after fatigue loading.	- A brief analytical explanation for strain sensing with a piezoelectric sensor was provided. - The PZT-5A powder was used as the piezoelectric material. - The initial vibration force test frequency was set at 100 Hz to evaluate piezoelectric paint performance. - The fatigue loading rate was approximately 10 Hz. - The cracks that were outside the sensor electrode area could not be detected by this sensor.
N. Lajnef et al. [113]	2011	STG	E	Pavement	- PZT and PVDF output power density. - Sensor output voltage as a function of cyclic load.	- MOS transistors managed data from the piezoelectric sensor. - A 62 mm × 10 mm × 28 μm PVDF piezoelectric sensor was used for energy harvesting and in a 1 Hz cyclic loading setup for designing a memory gates array. - In the temperature measurement test, a PZT disc embedded in a concrete beam underwent −10 to 40 °C temperature variations.
A. Alavi et al. [114]	2017	STG	E/S	Steel bridge	- Fatigue crack size vs. number of cycles. - Strain probability in a sensor array.	- For the analysis, a girder structurally similar to an existing highway steel bridge girder (I 96/M-52) in Webberville, Michigan, was chosen.
D. Kim et al. [115]	2019	STG	E	Single-lap joint steel	- The effect of different evaporation temperatures on the crystalline phase of PVDF films. - The effect of porosity and poling temperature on the piezoelectric constant of PVDF films. - S-N curve.	- PVDF films prepared from PVDF pellets via solvent casting in a 20 wt.% PVDF solution in dimethylformamide. - XRD analysis was conducted with a high-performance X-ray diffractometer using Cu Kα1 radiation (λ = 1.54 Å) at 40 kV and 40 mA. - Single-lap joint, consisting of S35C carbon steel adherents and biphenyl A diglycidyl ether-based epoxy resin with an amine-based hardener adhesive (10:8 *w*/*w* resin-hardener ratio), fabricated for lap shear strength measurement as a reference for fatigue loading conditions.
A. Ghaderiaram et al. [108]	2022	STG	E	Al dog-bone	- Fatigue strain.	- A 6061-T6 aluminum specimen was used for fatigue tests. - A soft PZT was used to measure strain. - A 3D-printed middleware was used to transfer the strain of the structure to the PZT sensor.

## Data Availability

The original contributions presented in this study are included in the article. Further inquiries can be directed to the corresponding author.

## Abbreviations

AE	Acoustic Emission
AIC	Akaike Information Criterion
CFRP	Carbon Fiber-Reinforced Polymer
CISS	Crack-Induced Second Source
CNN	Convolutional Neural Networks
COD	Crack Opening Displacement
CVS	Concrete Vibration Sensors
CWI	Coda Wave Interferometry
CWT	Continuous Wavelet Transformation
DAE	Deep Auto-Encoder
DFT	Discrete Fourier Transform
DI	Damage Index
DWT	Discrete Wavelet Transform
EDI	Envelope Damage Index
EMI	Electro-Mechanical Impedance
FCD	Fatigue Crack Diagnosis
FLM	Fatigue Life Monitoring
GW	Guided Waves
MBEJ	Modular Bridge Expansion Joints
MOS	Metal–Oxide Semiconductor
MOSFET	Metal–Oxide Semiconductor Field Effect Transistors
NDT	Non-Destructive Test
NI	Nonlinear Index
PCA	Principal Component Analysis
POD	Probability Of Detection
PTRFE	Poly-Trifluoro Ethylene
PVDF	Polyvinylidene Fluoride
PWAS	Piezoelectric Wafer Active Sensors
PZT	Lead Zirconate Titanate
RMSD	Root Mean Square Deviation
SHM	Structural Health Monitoring
SHMUS	SHM Ultrasound System
SNR	Signal-To-Noise Ratio
STFT	Short-Time Fourier Transform
STG	Strain Gauge
STSA	Symbolic Time Series Analysis
TC	Transmission Coefficient
TOA	Time Of Arrival
TOF	Time Of Flight
UGW	Ultrasonic-Guided Wave
UT	Ultrasonic Testing
XRD	X-Ray Diffraction
